# Normal pressure hydrocephalus: an update

**DOI:** 10.1590/0004-282X-ANP-2022-S118

**Published:** 2022-08-12

**Authors:** Carlos Eduardo Borges Passos-Neto, Cesar Castello Branco Lopes, Mauricio Silva Teixeira, Adalberto Studart, Raphael Ribeiro Spera

**Affiliations:** 1 Universidade de São Paulo, Faculdade de Medicina, Hospital das Clínicas, Departamento de Neurologia, Grupo de Neurologia Cognitiva e do Comportamento, São Paulo - SP, Brazil.

**Keywords:** Hydrocephalus, Normal Pressure, Dementia, Spinal Puncture, Hidrocefalia de Pressão Normal, Demência, Punção Espinal

## Abstract

Normal pressure hydrocephalus (NPH) has been a topic of debate since its introduction in publications. More frequent in the elderly population, it is characterized by gait disturbance, urinary urge incontinence and cognitive decline. Therefore, it is a clinical-radiological entity with relatively common findings for the age group, which together may have greater specificity. Therefore, its diagnosis must be careful for an adequate selection of patients for treatment with ventricular shunt, since the symptoms are potentially reversible. The tap test has a high positive predictive value as a predictor of therapeutic response, but a negative test does not exclude the possibility of treatment. Scientific efforts in recent years have been directed towards a better understanding of NPH and this narrative review aims to compile recent data from the literature in a didactic way for clinical practice.

## RESUMO

A hidrocefalia de pressão normal (HPN) é tema de debate desde sua introdução na literatura. Mais frequente na população idosa, caracteriza-se por distúrbio de marcha, urge-incontinência urinária e declínio cognitivo. Portanto, trata-se de uma entidade clínico-radiológica com achados relativamente comuns para a faixa etária, que em conjunto, podem ter maior especificidade. Sendo assim, seu diagnóstico deve ser criterioso para uma adequada seleção de pacientes para tratamento com a derivação ventricular, uma vez que os sintomas são potencialmente reversíveis. O tap test possui valor preditivo positivo alto preditor de resposta terapêutica, mas um teste negativo não exclui a possibilidade de tratamento. Esforços científicos nos últimos anos têm sido direcionados para melhor entendimento da HPN e essa revisão narrativa se propõe a compilar dados recentes da literatura de forma didática para a prática clínica.

## INTRODUCTION

Normal Pressure Hydrocephalus (NPH) is a matter of litigious debate since its introduction into scientific literature by the Colombian neurosurgeon Salomón Hakim in 1965. It is a syndrome defined by the presence of gait disturbance, urinary incontinence and cognitive decline (Hakim's triad), with progressive onset, radiological evidence of ventricular dilation and clinical improvement after shunting^1^ ^,^ ^2^. 

Classically, it is divided into secondary NPH (which occurs as a consequence of subarachnoid hemorrhage, trauma, brain tumors or infectious meningitis^1^ ^,^ ^3^) and idiopathic NPH (which reminds us of the unknown cause of that form^4^). However, recent debates have brought new ideas and challenged the classic concepts. An example is a theory that suggests the term neurodegenerative NPH, which infers NPH as a form of neurodegenerative pathology manifesting with hydrocephalus or as a disease continuum, which could explain the different results following treatment^5^. 

A relevant proportion of patients, when timely and properly diagnosed, presents significant benefit on neurological status and quality of life with ventricular shunt. Thus, clinical and scientific efforts in recent years have been directed to elucidate answers about this entity, its treatment, and to shed light on these gaps in our knowledge^6^ ^-^ ^8^. 

## EPIDEMIOLOGY

Due to the inherent difficulties in diagnosing NPH, an estimate of its prevalence is not easily feasible. The syndrome’s classic components may be unspecific in the elderly, and the need for lumbar puncture for diagnosis compromises the execution of population studies. Consequently, most studies on the prevalence of iNPH are based on hospitalar samples, which are often regionally and ethnically diverse, and, not least, with heterogeneous diagnostic criteria^9^ ^,^ ^10^. 

Since the 2000s, however, there has been an effort to create a diagnostic consensus^6^ ^-^ ^8^ ^,^ ^11^ ^,^ ^12^, which is expected to favor broader studies. Below, we comment on some available studies. 

In Japan, populational studies using the definition of possible NPH - based solely on radiological criteria (ventriculomegaly and disproportionate widening of cerebrospinal fluid spaces) - resulted in an average prevalence of NPH of 1.1% in the elderly^6^ ^,^ ^10^ ^,^ ^13^. 

Similar prevalence has been found in Western studies^14^. In a study with probable iNPH (with compatible clinical history and images, plus lumbar puncture) in Norway - the only public effort to recruit NPH patients in publications - the prevalence was 21.9/100,000, while the incidence was 5.5 per 100,000 inhabitants/year^15^. In a series of patients with cognitive decline, the prevalence varies between 3.5% and 10%^9^ ^,^ ^16^ ^-^ ^18^. In Brazil, Vale and Miranda (2002)^19^ observed that NPH represented 5.38% of cases of dementia in a tertiary hospital. Two other studies in Brazil have shown NPH as a relevant proportion of potentially reversible dementia (around 8%).^20^ ^,^ ^21^

When the motor symptoms are investigated, the numbers can be higher. NPH was responsible for 19% of suspected cases of parkinsonism in a German study^14^.

## PATHOPHYSIOLOGY

Classically defined as communicating hydrocephalus, NPH has normal opening pressure on lumbar puncture and a disproportionate ventricular dilation to the cortical atrophy degree.

As already mentioned, NPH is divided into an idiopathic and a secondary form. The latter is often related to subarachnoid hemorrhage, meningitis, intracranial tumors, traumatic brain injury, among other possible causes of poor cerebrospinal fluid reabsorption. It is assumed that these conditions lead to an inflammatory process of the arachnoid granulations, with reduced CSF reabsorption and alteration of the CSF flow dynamics, resulting in ventricular dilatation.

Regarding the so-called idiopathic NPH, the anatomopathological studies are heterogeneous. Among the findings described are thinning and fibrosis of the meninges and arachnoid membranes, inflammation of arachnoid granulations, vascular alterations, AD pathology, rupture of the ventricular ependymal and subependymal gliosis^6^. A minority of reported cases (10-20%) present increased head circumference, suggesting that it may be based on a congenital hydrocephalus that has become symptomatic over the years^22^. Other related rare causes are Paget's disease of bone affecting the base of the skull, mucopolysaccharidosis of the meninges and achondroplasia^23^.

Although no increase in intracranial pressure is observed during lumbar puncture, it is believed that there is a local pressure effect on the periventricular white matter^24^, with relevant importance of that pressure on the neuronal damage to periventricular structures, possibly responsible for the clinical syndrome. Brain perfusion studies in patients with hydrocephalus showed hypoperfusion in the periventricular white matter region, thalamus and basal ganglia, supporting the initial hypotheses. In addition, Arterial Spin-Labeling (ASL) MRI, which assess cerebral blood flow, have shown improvement in perfusion and clinical findings after a tap test^24^.

Also, apart from changes directly exerted by the local pressure, there is an increase in intracranial pulsatility pressure^24^, a reduction in brain compliance and vascular supply due to the involvement of terminal arterial vessels, with subcritical ischemia, but without infarction itself^24^. These alterations also lead to brain dysfunction, mainly subcortical dysfunction, highlighting the impairment of pathways related to the frontal lobe and its connections, preserving higher cortical functions such as gnosia, praxis, language and visuospatial ability in initial phases.

Recent associations between NPH and the glymphatic system (a CNS metabolic clearance pathway) may explain the frequent association with neurodegenerative comorbidities and further studies may elucidate the order of events in the pathophysiology of the entity^24^ ^,^ ^25^. 

## CLINICAL FEATURES

 The diagnosis is primarily based on history and neurological examination. The classic triad, described by Hakim, Adams and Fisher in 1965^2^, comprises gait disturbance, cognitive deterioration and urinary incontinence. The course is usually slowly progressive and the symptoms most commonly occur in that order, during a minimum period of three months^25^. The triad is not always completely present, especially in the early stages of the disease, and is not necessary for diagnosis^6^.

When considering the average age at diagnosis, it is important to keep in mind that these symptoms can be manifestations of many other conditions unrelated to NPH. Likewise, the patient with NPH may present other symptoms not described in the disease due to associated comorbidities, which makes the diagnosis challenging.

Gait disturbance is the most frequent symptom and, in most cases, the earliest. Labeled mainly as “apraxic gait” or “magnetic gait”, heterogeneity is observed among patients. Characteristically, the patient moves more slowly, with short steps, wide base and changes in direction using several steps, with the movement being fragmented or en bloc. There may be a posture of anterior inclination of the trunk, difficulty in climbing stairs and in performing transitional movements, such as sitting and standing up. The patient can more easily reproduce the gait movement in the sitting or supine position; however, he cannot perform this when in orthostasis^26^ ^,^ ^27^. Postural instability is common in these patients and can lead to frequent falls. Despite some characteristics similar to Parkinson’s gait, it should be noted that the patient does not present rigidity, festination, tremor of the upper limbs and facial hypomimia. Nor does he benefit from visual and auditory stimuli or cues as seen in Parkinson's disease^27^ ^,^ ^28^. The report of weakness and tiredness in the lower limbs is not infrequent, although the neurological examination does not show such alterations. In general, gait disturbance is the symptom that most responds to therapy, and is also the best evaluated in pre-surgical tests, as will be described below. It should be noted that gait disorders are frequent in the elderly population, especially those over 75 years of age, when they may be present in up to 20% of cases and even predict the risk of developing dementia^29^. 

Cognitive decline is secondary to frontal-subcortical pathway dysfunction, which mainly leads to a slowing of information processing speed and executive dysfunction. The patient may present with dementia itself, as well as mild cognitive impairment. Difficulty in sustained attention, abstract thinking, planning, decision making, and problem solving is observed^2^ ^,^ ^25^ ^,^ ^29^. Memory is relatively preserved in the initial phases, but its alteration in the following phases is variable. As a behavioral change, loss of volition and apathy can be observed. Depressed mood is prevalent. 

Urinary symptoms are defined as an uninhibited neurogenic bladder, with urgency, increased frequency, with or without incontinence in the early stages. This type of finding should be well defined in history, as it is also a very prevalent symptom in the population aged over 60^29^. The presence of other cortical symptoms, such as language alteration, apraxia, agnosia, prosopagnosia, and loss of visuospatial ability suggest another diagnosis, or a comorbidity. Lateralized or dimidiated symptoms are atypical for the disease, as are rigidity, tremor, bradykinesia, spasticity, hyperreflexia, and other signs of upper or lower motor neuron involvement, especially in the early stages. Rapidly progressive course is uncommon, although there are cases described in a Greek cohort^30^.

The two most used NPH guidelines are the International^8^ and the Japanese NPH Society(6).

## DIFFERENTIAL DIAGNOSIS

The diagnosis of NPH is rarely performed in isolation - it requires a broad investigation to rule out its multiple possibilities of differential diagnosis. The elderly patient may have several comorbidities that can lead to NPH cardinal symptoms. Cervical or lumbosacral spondylosis, large joint osteoarthritis, peripheral neuropathy, vestibular dysfunction, reduced muscle mass and even visual impairment are examples of reported etiologies for gait disturbance. Similarly, neurologic and non-neurologic causes of urinary incontinence should be considered and evaluated, such as urinary tract infection, prostatic hypertrophy, myelopathy, pelvic organ prolapse, bladder outlet obstruction and side effects of medication.

However, the clinician should be aware of the main neurodegenerative diseases that present signs and symptoms that can lead to a misdiagnosis of PNH, especially when cognitive impairment is a prominent symptom. The main features of each of these are:

### Disease with Lewy bodies

Cognitive decline with predominant change in ability, visuospatial and executive dysfunction, complex visual hallucinations, Parkinsonism, and fluctuating consciousness. Intolerance to neuroleptics and good response to cholinesterase inhibitors.

### Parkinson's disease

Parkinsonism evident in the early stages and with unique characteristics (rest tremor, cogwheel rigidity, bradykinesia, and Parkinson gait with postural instability), markedly asymmetrical, an important response to levodopa that may

lead to dyskinesia and cognitive manifestation of late evolution.

### Vascular dementia

Asymmetric findings, focal deficits, early pyramidal changes (exalted reflexes, spasticity and presence of Babinski's sign), acute onset and evolution in steps or cognitive stability when the disease is controlled.

### Vascular Parkinsonism

Presence of Parkinsonian symptoms that predominate in the lower limbs and gear change. Neuroimaging investigation demonstrates microangiopathy and/or ischemic lacunar lesions in the periventricular and basal ganglia regions.

### Progressive supranuclear palsy

May have early impairment of gait and is associated with frequent falls. However, it evolves with Parkinsonian symptoms with axial rigidity, alteration of extrinsic ocular motricity (initially vertical), cervical and facial dystonia, which lead to expression of consternation characteristic of the disease (omega sign).

### Alzheimer's disease

May be associated with NPH, especially when the condition presents prominent amnestic changes and deficits suggestive of cortical dysfunction, such as loss of visuoperceptual and visuospatial functions, language alteration and apraxia. A change of gait is not common in the early stages of AD alone.

## NEUROIMAGING

### Brain CT and MRI

Neuroimaging showing ventriculomegaly is essential for diagnosis, as well as for excluding NPH mimics.

CT is able to show enlargement of lateral and third ventricles, as well as other typical findings of the disease; however MRI is more accurate. Several findings are described and together they help to define the diagnosis.

**Figure 1.**
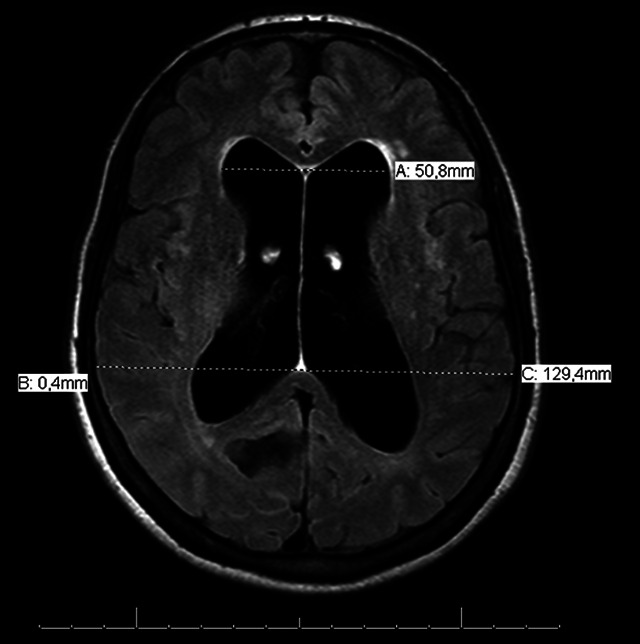
Axial view on FLAIR (*Fluid-Attenuated Inversion Recovery*) sequence showing important ventriculomegaly and high Evans Index, calculated by the reason of bilateral ventricular frontal horns greatest diameter over the greatest skull diameter (estimated as > 0,3 on this example).

**Figure 2.**
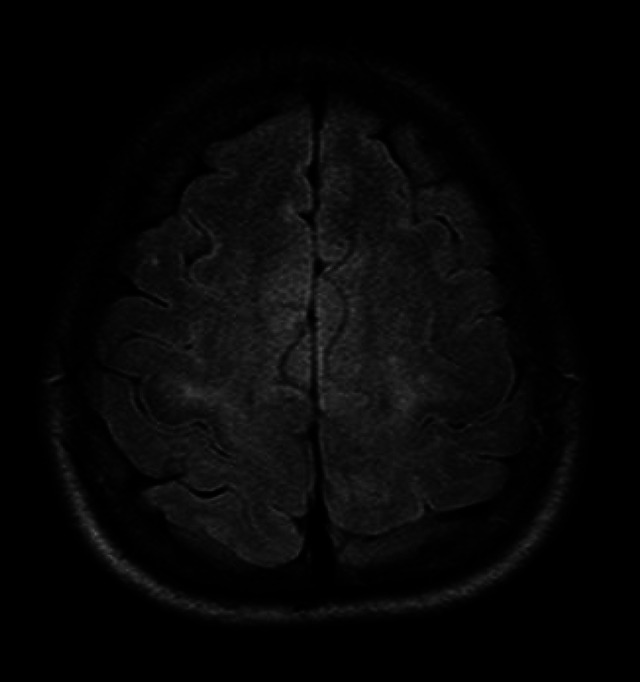
Axial view on the FLAIR sequence (*Fluid-Attenuated Inversion Recovery*) showed narrow cortical sulci on the high convexity of the frontoparietal areas.

**Figure 3.**
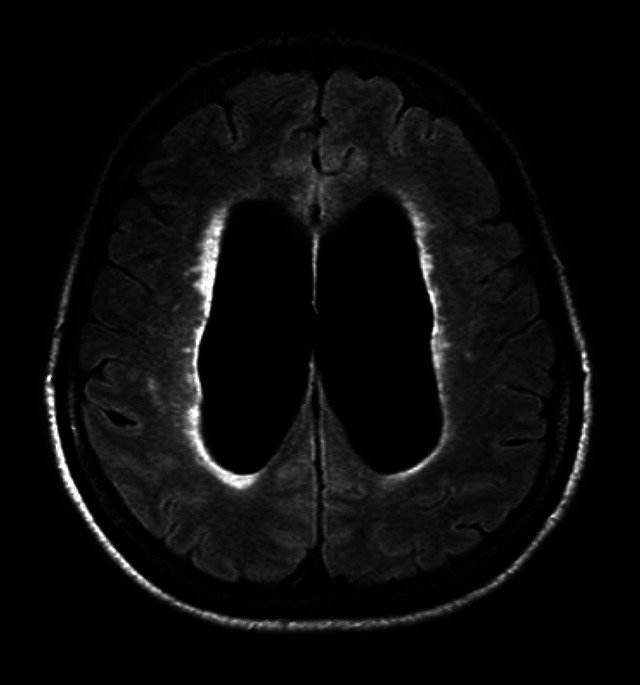
Axial view on the FLAIR sequence (*Fluid-Attenuated Inversion Recovery*) showing hipersignal over the lateral ventricle margins, suggesting ependimary transudate of the CSF.

**Figure 4.**
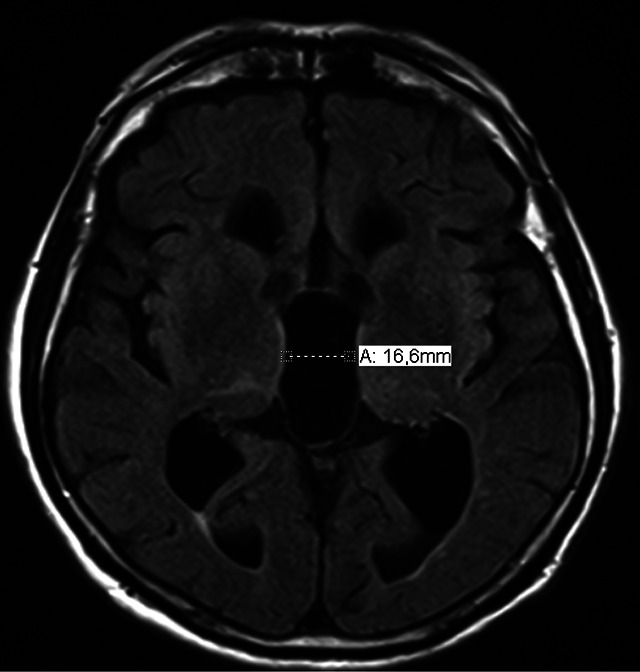
Axial view on the FLAIR sequence (*Fluid-Attenuated Inversion Recovery*) showing prominent third ventricle dilation.

**Figure 5.**
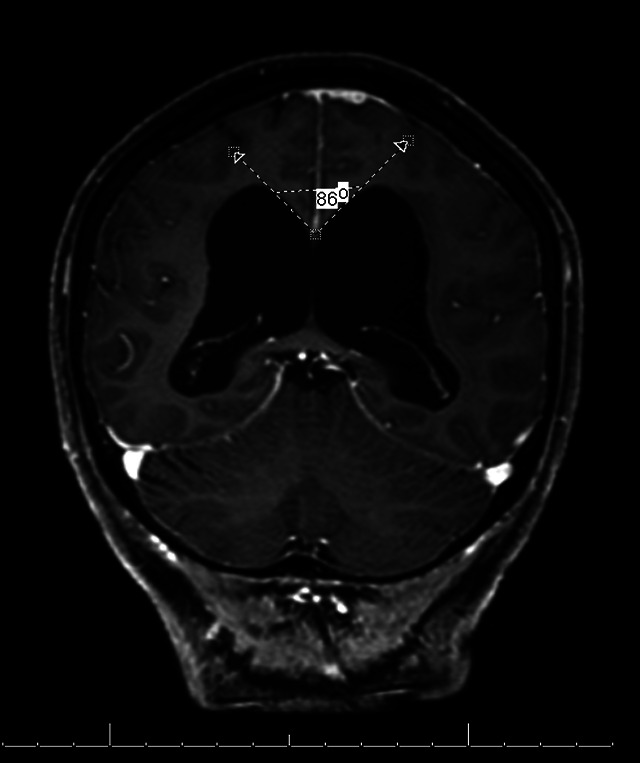
Coronal view on the T1 sequence with contrast showing acute callosal angle.

**Figure 6.**
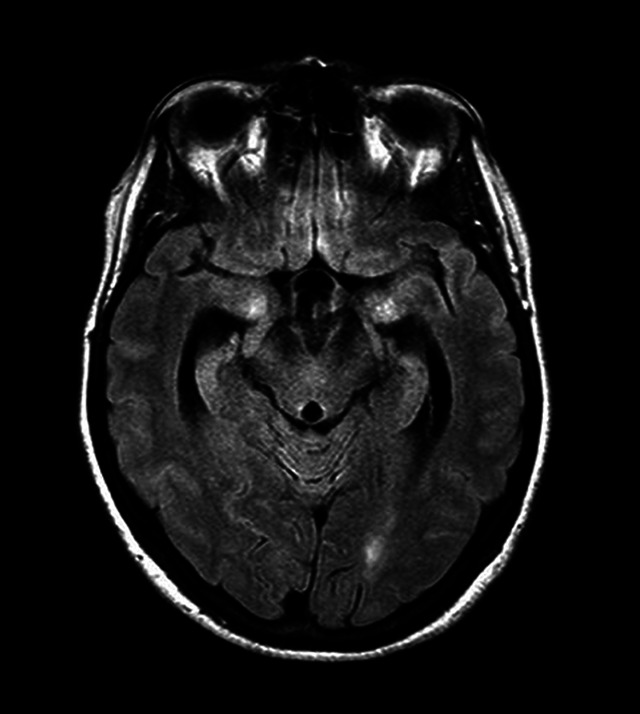
Axial view on the FLAIR sequence (*Fluid-Attenuated Inversion Recovery*) showing cerebral aqueduct dilation, characterized as *flow-void*.

Evans index, one of the first signs described, is able to diagnose and quantify ventriculomegaly. The calculation is made through the ratio between the largest diameter between the frontal horns of the lateral ventricles over the largest diameter of the cranial vault cavity observed in the same axial section. A ratio greater than 0.3 indicates ventricular dilatation^2^ ^,^ ^6^ ^,^ ^25^ ^,^ ^29^ (Figure 1). It should be noted that this finding is not specific or pathognomonic of NPH. A recent study suggests the adoption of different values ​​depending on the age group and sex of the patients: 

- Flattening of the cortical sulci in high convexity with widening of the sylvian fissure and ventricular dilation can be observed^6^ ^,^ ^25^ (Figure 2);
- Marginal hypersignal to the lateral ventricles on T2-weighted or FLAIR sequences due to ependymal CSF transudation (Figure 3);
- Dilation of the third ventricle^23^ (Figure 4);
- Reduction of the callosal angle in the coronal section^31^ ^,^ ^32^ (Figure 5);
- Increased CSF flow in the Sylvius aqueduct associated with flow void^25^ ^,^ ^33^ (Figure 6) are described and considered debatable predictors of the response to ventricular shunt^32^

There used to be a supposition that NPH patients had inverted CSF flow in the cerebral aqueduct, but this finding was not confirmed in a recent study using phase contrast MRI. However, an increase in the variability and pulsatility of the CSF flow in the aqueduct was demonstrated, with a reduction in the flow pulsatility in the cervical region. These findings may be incorporated in the future to aid diagnosis and assessment of the post-operative prognosis^33^. CSF flow obstruction, such as aqueduct stenosis or Chiari malformation^6^ should not be observed.

Finally, it is important to remember that ventriculomegaly does not imply NPH. Iseki et al. (2009) observed that 1% of the elderly in their sample had findings compatible with NPH on MRI without any symptoms. However, 25% of these developed clinically were suggestive of NPH during follow-up, suggesting that ventricular dilatation may anticipate the development of symptoms^3^ ^4^. 

### SPECT and PET-CT

SPECT and PET-CT can aid the diagnosis, revealing hypometabolism in the periventricular and frontal regions, respectively.

PET-PiB plays a minor role in the differential diagnosis, considering the advanced mean age of these patients, 32% of the patients demonstrate amyloid deposits on examination^29^.

### Magnetic resonance spectroscopy

Some parameters of magnetic resonance spectroscopy (MME) have been suggested as potentially useful in the diagnosis and follow-up of patients with NPH (Table 1).

**Table 1:** Potential parameters of magnetic resonance spectroscopy (MME) for diagnosis and follow-up of patients with iNPH

Parameter	Finding
Lactate (diagnosis)	Peak at the lateral ventricle^34^
Proton MRS (follow-up)	Lower N-AcetylAspartate/Creatine ratio in iNPH patients with improvement after shunt^35^ ^,^ ^36^, one study failed to find similar results^37^

Currently, the value of spectroscopy for diagnosis and follow-up of response in iNPH is controversial^6^.

### Radioisotope cisternography

Cisternography involves the injection of a radioisotope or contrast into the subarachnoid space, via lumbar puncture, with subsequent acquisition of serial images to observe the cerebrospinal fluid progression. Gadolinium MRI cisternography is the most modern method^40^, but the most used is hybrid SPECT/CT imaging with 99Tc or 111In-DTPA radioisotopes^41^ ^,^ ^42^. In iNPH, interpretation is usually done after 24-48h. Typical NPH findings are intraventricular reflux and radioisotope stasis on the brain surface^41^. Although radioisotope cisternography (CRI) was considered necessary for the diagnosis of NPH (secondary or idiopathic), performing this test did not add diagnostic accuracy in patients with clinical and cranial CT compatible with iNPH^43^. Indeed, some studies show that the CRI is inferior to the tap test or external lumbar shunt as a predictor of shunt response^44^. There is not enough evidence to determine its value in the selection of good candidates for shunt among patients and since it is an invasive test, CRI is currently not indicated for diagnosis of iNPH^6^ ^,^ ^7^ ^,^ ^11^, although it may be useful to investigate CSF obstruction^6^. 

### Confirmatory tests and predictors of treatment efficacy

The treatment of NPH is surgical and carries a potential risk of complications, therefore, tests that can predict the therapeutic response after surgery are indicated, in addition to detailed clinical analysis and comorbidities.

Tap Test (TT) - it is considered important as a pre-surgical evaluation and helps in the diagnosis when the response is positive. It is simple, easy to perform, with low complication rates^45^, and is performed in a hospital regime without hospitalization or the need for a surgical center.

Lumbar puncture is performed in the usual way, with the withdrawal of 30-50 ml of CSF. Before and after the procedure, the patient is evaluated for his cognition and gait. In this respect, there is a lack of standardization in the literature regarding the evaluation methods, as well as regarding the evaluation time after the puncture, and the studies are heterogeneous^45^. In general, gait is evaluated through cadence, speed, step size and change of direction. Time Up and Go can be used for quantification, as well as other scales, depending on the service experience. Gait is the symptom that most responds to the Tap Test.

Regarding cognitive assessment, the well-known Mini-Mental State Examination can be used, with the aim of comparing pre- and post-puncture and posterior longitudinal follow-up. The clock drawing test and phonemic and semantic verbal fluency can be used to complement cognitive assessment. Moreover, several other assessment methods can be used^46^. The average post-procedure evaluation time is between 30 minutes and four hours, although some articles mention up to 24-48 hours or even a week. Gait improvement was prevalent, but cognitive response occurred in only two studies in the meta-analysis. Improvement in urinary continence was not observed. The duration of the response was also variable^45^.

A recent systematic review^45^ demonstrated that TT has a high positive predictive value, generally above 90% (73-100%). However, the negative predictive value is heterogeneous and tends to be low - less than 20% in some studies (18-50%). The overall accuracy was 62%. These data indicate that the patient may not respond to the pre-operative procedure, although when the diagnosis is presumed to be probable and there is a high suspicion of response to shunting, shunting can still be considered despite the TT result. Other possibilities would be the repetition of the TT and/or sequential performance in three days, as well as the use of another evaluation method.

### Lumbar infusion resistance test

The CSF infusion test (CSF-IT)^47^ is based on the disturbance of CSF hydrodynamics observed in iNPH and is considered a central aspect of its pathophysiology. This change is reflected in an increase in resistance to fluid infusion in the subarachnoid space. CSF-IT is performed by injecting saline or artificial CSF into the subarachnoid space and measuring the initial and final pressures (plateau) and then calculating resistance parameters. Despite this pathophysiological basis, the utility of CSF-IT as a predictor of shunt response has been difficult to establish, due to conflicting results^15^ ^,^ ^46^. 

### CSF analysis

CSF analysis is often normal, notably opening pressure, and can be used to rule out other pathologies. Regarding biomarkers, a reduction in proteins derived from the amyloid precursor (Aß38, Aß40 and Aß442) stands out, in contrast to the decline specific to the Aß42 found in the AD^33^. 

### Continuous monitoring of intracranial pressure

 Despite the name normal pressure hydrocephalus, transient increases in intracranial pressure are seen in the patients, especially during the night. Among the related alterations, the increase in the frequency of B waves is marked, particularly during REM sleep^48^. The occurrence of B waves was associated with a greater response rate to shunting^49^, but this correlation has not been confirmed in subsequent studies^50^. New studies are necessary to establish the utility of the continuous monitoring of intracranial pressure in the evaluation of patients with suspected iNPH.

### External lumbar drainage

 External lumbar drainage consists of the constant removal of CSF from the lumbar subarachnoid space, with a drainage system connected to a valve and a collection bag. As the point of the procedure is to predict the response to the shunt, the drainage rate is similar to that of the ventriculoperitoneal shunt with a medium-pressure valve (10 ml/hr)^51^. The drainage is maintained for three to five days. As in the tap test, gait and cognition evaluations should be performed before and after the procedure.

 A positive response to the external lumbar drainage has been associated with an increased chance of responding to the shunt, but the negative predictive value is low^52^. In a class III study, more than 80% of the patients with a positive external lumbar drainage responded to shunting, using the gait as the main parameter. However, one patient with a negative test (of a total of three) also had a positive response to shunting. Another study showed that, although the response to the tap test and external lumbar drainage was suggestive of a good response to the shunt, about half of patients with a negative response benefited from surgery as well, indicating a high false-negative rate^48^.

 Some factors limiting the routine application of this procedure are its invasive profile (with risks of hematoma and infection) and the need for hospitalization, since it is performed in the operating theater.

## TREATMENT AND OUTCOME

Ventricular shunting is the treatment of choice in iNPH but, in this subset of patients, the experience with the shunt can be replete with some difficulties, such as variable or short-term responses, as well as the risk of complications^21^ ^,^ ^52^ ^-^ ^56^. Because of this, patients who are candidates for ventricular shunting should also be assessed with regard to comorbidities that could influence the outcome or increase the procedure risk. 

The shunt can be done either to the peritoneum (ventriculoperitoneal) or to the atrium (ventriculoatrial), with no differences in the prognosis between the two. In those with contraindications to ventricular shunting, lumboperitoneal (LP) shunting can be performed^53^ ^-^ ^56^. Following the SINPHONI-2 trial^57^, there seems to be similar efficacy between LP and ventriculoperitoneal and ventriculoatrial shunting. However, although it is less associated with infection than ventriculoperitoneal shunting, it has greater risk of system malfunction^6^. 

There are different types of valves used. The fixed-pressure valve was most used in the past. Nowadays, the new adjustable valves are more appropriate, mainly because of fewer associated complications, such as subdural fluid collections and corrective surgery^58^ ^-^ ^60^. Overall, the risk of severe complications has been estimated at 10-11%^57^. 

It is not clear if the effect of multiple comorbidities can negatively influence the response after the procedure^7^. Older age by itself is not considered a predictor of bad response. Considering the risks, patients with nonspecific symptoms and without benefit of the tap test shouldn't be considered eligible for shunting, nor should those with asymptomatic ventriculomegaly.

 The immediate response to the ventricular shunting is good. In six and 12 months after the procedure, there is evident clinical benefit in 90 and 80% of the patients, respectively^7^ ^,^ ^58^. 

 The evaluation parameters of response are not standardized, but in general they include a disability scale, such as the modified Rankin scale, and/or an evaluation of the severity of the symptoms of iNPH. The most used scales attribute points to gait, urinary continence and cognition. Gait is usually the alteration that is most sensitive to shunting, with around 83% of the patients presenting improvement in the gait evaluation (number of steps and time in seconds to walk 10 meters)^7^.

 There are no studies investigating the long-term outcome of patients not eligible for shunting, and there is still no evidence that the temporary benefit of ventricular shunting can be sustained over years^6^.

In conclusion, NPH is a relatively prevalent medical condition, especially in the older age group. Gait disturbance is the most common and usually the first to manifest itself. It is also a reversible cause of dementia, therefore the correct diagnosis should be pursued in order to properly indicate the therapy. Although the triad: gait disturbance, cognitive impairment and urinary incontinence is well known, it is not present in many patients and shouldn’t be anticipated in treatment of the patient.

Neuroimaging is essential to determine the typical findings of NPH, such as ventriculomegaly, DESH and narrow callosal angle. Nonetheless, even with clinical and radiological features suggesting NPH, it is important to objectively evaluate the clinical response to other tests, such as the tap test, before indicating shunting. In the suggestive cases of NPH with good response to the test, shunting (ventricular or lumbar) should be performed after careful discussion with the patient and family regarding the possibility of improvement of the symptoms and quality of life, but also the comorbidities, risks associated and the possibility of a transient response to the treatment. In patients with no response to the test but with suggestive features of HPN, it should be personalized as to whether the patient should or should not receive the invasive treatment.
